# The neurovascular coupling basis of cognitive impairment in patients with H-type hypertension: a cross-modal study using resting-state fMRI and arterial spin labeling

**DOI:** 10.3389/fmed.2026.1918075

**Published:** 2026-08-26

**Authors:** Chun Zhang, Qiaomei Zhao, Caihong Song, Haozhi Chen, Wei Xing

**Affiliations:** 1 Department of Radiology, Huai'an Maternity and Child Healthcare Hospital, Huai'an, Jiangsu, China; 2 Department of Laboratory Medicine, The Fifth People's Hospital of Huai'an, Huai'an, Jiangsu, China; 3 Department of Rehabilitation Medicine, Huai'an Maternal and Child Health Care Center, Huai'an, Jiangsu, China; 4 Department of Radiology, Third Affiliated Hospital of Soochow University, Changzhou, Jiangsu, China

**Keywords:** ASL, homocysteine, H-type hypertension, neurovascular coupling, rs-fMRI

## Abstract

**Objectives:**

To investigate local and global neurovascular coupling (NVC) alterations in H-type hypertension (HHT) and their relationships with homocysteine (Hcy) levels and cognitive deficits and whether they reflect cognitive and disease severity.

**Methods:**

Fifty-eight HHT patients and fifty normotensive healthy controls, matched for demographics and comorbidities and meeting MRI quality control standards, were included. Data collected included demographic, clinical, biochemical, cognitive, and multimodal MRI measures.

**NVC analysis:**

After preprocessing and spatial normalization to MNI space, rs-fMRI and ASL data were used to calculate indices of neural activity—ALFF, fALFF, ReHo, FCD—and cerebral blood flow (CBF). Using the AAL atlas and partial correlation, global and regional NVC values were calculated.

**Results:**

HHT patients exhibited elevated Hcy and cognitive impairment (lower MoCA/MMSE). Imaging revealed DMN alterations, increased basal ganglia ALFF, and hypertension-related CBF redistribution (decreased parietal/posterior flow; increased frontal/hippocampal flow). Global and local NVC were generally reduced, particularly in occipital and temporal regions. Crucially, global NVC correlated negatively with systolic BP and Hcy, and positively with cognitive scores.

**Conclusions:**

HHT patients exhibit reduced global and altered regional NVC—especially in somatosensory and default mode networks. Because the design is cross-sectional, these findings provide multimodal MRI evidence of how altered neurovascular coupling is associated with HHT—highlighting the neural mechanisms underlying cognitive impairment in HHT.

## 1 Introduction

H-type hypertension (HHT) denotes essential hypertension accompanied by hyperhomocysteinemia, conventionally defined as a plasma homocysteine (Hcy) concentration ≥ 10 μmol/L. It is not a minor subgroup: this phenotype accounts for a large proportion of hypertensive adults in China, and it carries a stroke and cognitive risk in excess of that conferred by elevated blood pressure alone (1–3). The clinical question that follows is what distinguishes the brain of a patient with HHT from that of a patient with uncomplicated hypertension, and at which level of organization that difference is best measured.

H-type hypertension causes multiple damages to the cerebrovascular system, brain parenchyma, and cerebral metabolism, significantly increasing the risk of cognitive impairment (1–5). Neuroimaging studies have offered novel insights into how HHT influences both brain structure and function. For instance, Hcy, known for its neurotoxicity, promotes the deposition of β-amyloid (Aβ) plaques and the formation of neurofibrillary tangles, leading to cortical thinning, increased white matter hyperintensities, and reduced gray matter volume, by damaging endothelial cells, it exacerbates cerebral small vessel disease and accelerates brain aging, thereby being recognized as an independent risk factor for age-related cognitive decline (6–8). Correspondingly, primary hypertension contributes synergistically to brain damage, including relative cortical hypoperfusion, widespread gray matter atrophy, alterations in the default mode network and functional connectivity, as well as disruptions in the frontoparietal network and corresponding impairments in white matter integrity (9–13).

Multimodal MRI leverages its advantages in imaging sequences to enable cross-modal studies of brain structure and function. For instance, resting-state functional MRI (fMRI) based on the blood-oxygen-level-dependent (BOLD) signal can measure and reflect low-frequency spontaneous neural activity in the human brain, and has been widely used to detect large-scale spontaneous functional architectures and local neural activity. Another functional imaging technique at a more specific level is cerebral blood flow (CBF) imaging based on arterial spin labeling (ASL), which has been extensively applied to quantify both global and regional CBF. The integration of these two techniques has emerged as a prominent research theme in neuroimaging in recent years, referred to as NVC. Each index carries a distinct physiological meaning: ALFF and fALFF quantify the amplitude of spontaneous low-frequency BOLD fluctuation and thus index regional neural activity; ReHo quantifies the temporal synchrony of a voxel with its immediate neighbors; global and local functional connectivity density (gFCD, lFCD) quantify the number of long- and short-range functional connections of each voxel; and ASL-derived CBF quantifies regional perfusion, the supply term that cerebral autoregulation adjusts to meet local metabolic demand. Because the BOLD-derived indices reflect neural activity and the ASL-derived map reflects the perfusion that such activity elicits, the spatial correspondence between the two within an individual brain provides a non-invasive, macroscale surrogate of neurovascular coupling (14).

The main principle of NVC analysis is based on the neurovascular unit (NVU) (14–16). The human brain's NVU exhibits temporal and spatial synergy in terms of oxygen supply and metabolic demand. This synergy, also termed “neurovascular coupling,” ensures that dynamic energy metabolic demands are precisely matched to local neural activity, thereby facilitating efficient and synchronized neural processes and supporting normal cognitive function (17–20). Alterations in NVC have been associated with normal aging, cerebral small vessel disease, and end-stage renal disease (21–25).

Two insults converge on the neurovascular unit in HHT. Chronic pressure load drives inward eutrophic remodeling of penetrating arterioles, impairs myogenic autoregulation and shifts the autoregulatory curve to the right, so that perfusion becomes more sensitive to any fall in driving pressure. Homocysteine acts on the same unit by a different route: it reduces nitric oxide bioavailability, generates oxidative stress in endothelial cells, acts as an agonist at NMDA receptors and increases blood-brain barrier permeability, with secondary injury to pericytes and astrocytic end-feet. Since each insult degrades the match between neural demand and vascular supply rather than either term alone, a coupling measure, rather than perfusion or spontaneous activity in isolation, is the read-out that should be most informative in this disease.

Animal studies suggest that hypertension is associated with macro-level reductions in cerebral blood flow and disruption of the local microvascular environment, such as damage to endothelial cells and the blood-brain barrier (26). Human neuroimaging research indicates that hypertensive patients exhibit local gray matter atrophy, impaired white matter integrity, and alterations in resting-state spontaneous brain activity (27). This body of evidence suggests that HHT may represent a form of cerebrovascular disease characterized by impairment of the NVU—a hallmark feature of neurovascular decoupling. Notably, varying degrees of neurovascular decoupling have been reported in patients with hemorrhagic and ischemic stroke, as well as in neurodegenerative disease (21, 22, 27–33).

HHT represents the dual stresses of hypertension and elevated homocysteine (Hcy), both of which have been reported to alter the NVU, cerebral perfusion pressure, and spontaneous neural activity. However, due to the lack of non-invasive human brain measurement techniques, it remains unclear how HHT affects the NVU or large-scale, macroscale NVC. Therefore, this study aims to apply a novel methodological approach to explore: (1) whether HHT alters NVC; (2) which brain regions exhibit altered NVC and to which large-scale brain networks these regions belong; and (3) how such alterations correlate with cognitive scale assessments.

## 2 Material and methods

### 2.1 Participants

The HHT patients included in this study were all newly diagnosed, first-visit cases identified at the hospital. They were recruited immediately after diagnosis for study enrollment. Following data collection, most patients had received conventional medication or had actively adjusted their lifestyles. A total of 62 HHT patients and 52 normotensive subjects were initially recruited as healthy controls. After visual quality control and resting-state fMRI preprocessing, four patients and two controls were excluded. One patient was excluded because of incomplete imaging data, while three patients and two controls were excluded because of excessive head motion during fMRI acquisition. Consequently, the final analysis included 58 HHT patients and 50 normotensive controls. All participants provided written informed consent. The study protocol was approved by the Human Research Ethics Committee of the Third Affiliated Hospital of Soochow University and complied with the ethical principles outlined in the Declaration of Helsinki.

All participants underwent diagnosis and examination by qualified physicians. Each subject received a comprehensive neurological evaluation, blood tests, and neuropsychological assessments.

Inclusion criteria for HHT patients were: (1) age between 18 and 65 years; (2) right-handedness; (3) Met the diagnostic criteria for HHT (serum Hcy level ≥10 μmol/L and clinical systolic blood pressure ≥140 mmHg and/or diastolic blood pressure ≥90 mmHg); (4) no current antihypertensive treatment; (5) No contraindications for MRI examination; Inclusion criteria for the healthy control group were: healthy volunteers aged 18–65 years.

Exclusion criteria for all participants included: (1) any history of cardiovascular or cerebrovascular events; (2) significant history of cerebral infarction or cerebral hemorrhage; (3) major systemic diseases (including psychiatric disorders, renal failure (serum creatinine >176 μmol/L), liver impairment (AST or ALT >40 IU/L), coronary heart disease, congestive heart failure, or a history of malignant tumors); (4) other structural brain abnormalities that may affect cognitive function; (5) education level lower than 6 years.

### 2.2 Demographic, clinical, and cognitive data acquisition

The following diagnostic criteria were applied in this study: hypertension was defined as a resting seated systolic blood pressure ≥140 mmHg and/or diastolic blood pressure ≥90 mmHg, or a documented history of hypertension. Hyperhomocysteinemia was defined as a serum Hcy level ≥10 μmol/L.

Blood pressure was measured by two professionally trained researchers using a validated Omron electronic sphygmomanometer fitted with an appropriately sized cuff according to the participant's arm circumference. Participants rested in a seated position for at least 5 min before measurement, with their backs supported, feet flat on the floor, legs uncrossed, and the arm supported at heart level. They were instructed to avoid caffeine intake, smoking, and strenuous exercise for at least 30 min before measurement. Three consecutive measurements were obtained from the same arm at 1–2 min intervals, and the mean of the second and third readings was recorded. Hypertension was defined in accordance with the 2018 Chinese Guidelines for the Management of Hypertension, based on elevated blood pressure measurements obtained on separate occasions and/or a documented history of hypertension. The device was regularly maintained and calibrated according to the manufacturer's recommendations.

In addition to blood pressure data, we collected demographic characteristics and past medical history information (Table 1). Neurocognitive function was assessed in all participants using internationally recognized comprehensive cognitive evaluation tools: the Mini-Mental State Examination (MMSE) (34) and the Montreal Cognitive Assessment (MoCA) (35).

**Table 1:** Demographic, clinical, and cognitive assessment data.

Demographic data	HHT (*n* = 58)	HC (*n* = 50)	Statistic t/χ^2^ (*p*)
Age, mean (SD), y	48.96 ± 6.69	47.34 ± 6.90	1.238 (0.219)
Sex: Male/Female	32/26	25/25	0.288 (0.591)#
Education, mean (SD), y	11.304 ± 2.558	11.780 ± 2.957	−0.882 (0.380)
Smoking: *n* (%)	27/31	19/31	0.803 (0.370)#
Systolic BP, mean (SD), mmHg	153.241 ± 7.828	118.100 ± 11.427	18.348 (< 0.001^***^)
Diastolic BP, mean (SD), mmHg	99.379 ± 10.574	74.721 ± 7.570	14.065 (< 0.001^***^)
Hcy (μmol/L)	24.073 ± 11.698	10.340 ± 2.858	8.646 (< 0.001^***^)
LDL	2.775 ±0.653	2.732 ± 0.669	0.335 (0.739)
HDL	1.080 ± 0.282	1.220 ± 0.289	−2.491 (0.014)^*^
Triglycerides	2.120 ± 1.012	1.647 ± 1.341	2.016 (0.047)^*^
Total cholesterol	4.826 ± 0.924	4.897 ± 0.983	−0.378 (0.706)
Fasting blood glucose	5.771 ± 1.587	5.233 ± 1.304	1.904 (0.060)
HbA1c	6.443 ± 1.748	6.074 ± 0.944	1.195 (0.236)
Creatinine	72.168 ± 16.162	64.828 ± 13.060	2.572 (0.012)^*^
Urea nitrogen	5.1768 ± 1.6099	5.1292 ± 1.200	0.173 (0.863)
CRP	6.9107 ± 8.121	6.4836 ± 10.025	0.169 (0.866)
MMSE	29.018 ± 1.168	29.740 ± 0.527	−4.176 (< 0.001^***^)
MoCA	27.858 ± 1.354	28.900 ± 0.974	−4.585 (< 0.001^***^)

Table shows mean (standard deviations) for all variables for hypertensive subjects and normotensive controls in this study. LDL, low-density lipoprotein; HbA1c, Glycosylated Hemoglobin, Type A1C; # represents the chi-square test. ^*^p < 0.05, ^**^p < 0.01, ^***^p < 0.001.

The MMSE and MoCA were administered by a trained assessor who took no part in MRI acquisition, image processing or statistical analysis and who did not have access to plasma Hcy results at the time of testing. Because blood pressure was measured earlier in the same clinical visit, complete blinding to group allocation could not be guaranteed; both instruments are fully structured, with fixed items and objective scoring rules, which limits the scope for assessor bias.

### 2.3 MRI data acquisition

MRI data acquisition methods were consistent with those previously described. All MRI data were collected using a German Siemens 3.0 T MAGNETOM Verio MRI scanner (located at the Third Affiliated Hospital of Soochow University). The following scanning sequences were employed in this study:

(1) Whole-brain T1-weighted structural imaging: Acquired using a magnetization-prepared rapid gradient echo (T1-MPRAGE) sequence. Parameters: sagittal three-dimensional acquisition; slice thickness = 1 mm; time of repetition (TR) = 1,900 ms; time to echo (TE) = 2.52 ms; inversion time = 900 ms; flip angle = 9°; matrix size = 256 × 256. (2) Resting-state fMRI imaging: Acquired using a T2^*^-weighted gradient-echo echo-planar imaging (EPI) sequence. Main parameters: TR/TE = 2,000/30 ms; flip angle = 90°; number of excitations (NEX) = 1; field of view (FOV) = 220 mm; raw phase-encoding dimension Ny = 64; slice thickness = 4 mm; total scanning duration = 8 min. (3) Perfusion imaging: Arterial spin labeling (ASL) sequence was used to obtain relative cerebral blood flow (rCBF) maps. Parameters: FOV = 220 mm; slice thickness = 6 mm; TR/TE = 2,500/11 ms; phase-encoding dimension sampling matrix Ny = 61; NEX = 50; flip angle = 90°. The sequence included pre-saturation/fat suppression and background suppression modules (PFP/SAT1/FS) to suppress signals from non-perfused tissues and reduce noise and artifacts. (4) Diffusion tensor imaging (DTI): Acquired using a two-dimensional sequence. Parameters: slice thickness = 3 mm; slice gap = 1.875 mm; TR/TE = 3.8/0.102 s; flip angle = 90°; matrix size = 128 × 128. The DTI sequence utilized 64 diffusion directions (b = 1,000 s/mm^2^) and one b = 0 s/mm^2^ acquisition, implemented with a GRAPPA-accelerated single-shot spin-echo EPI sequence. In addition, conventional MRI sequences were also acquired, including T2-3D and susceptibility-weighted imaging (SWI), to rule out significant lesions, congenital malformations, or other abnormalities. Detailed parameters of these routine clinical MRI sequences are not elaborated here.

### 2.4 Resting-state fMRI data preprocessing

Resting-state fMRI image preprocessing was performed using SPM12 and the DPABI toolkit (DPABI version 4.5, http://rfmri.org/dpabi) on the MATLAB R2018b platform. After quality control and screening, all images were subjected to the following preprocessing steps:

(1) Format Conversion: The original DICOM images were first converted to NIFTI format. The initial 10 time points of the resting-state fMRI data were discarded to allow for signal stabilization. (2) Slice Timing and Head Motion Correction: Slice timing and head motion corrections were applied. For head motion correction, framewise displacement (FD) was calculated in addition to the conventional exclusion criteria (translation >2.0 mm or rotation >2°). FD values were used as covariates for micro-motion regression. (3) Anatomical Coregistration: The T1-weighted anatomical images, after brain extraction and alignment to the anterior commissure–posterior commissure (AC-PC) line, were coregistered with the functional images to compensate for the lower resolution and potential signal loss in the functional data. (4) Spatial Normalization: The T1 anatomical images were segmented into gray matter (GM), white matter (WM), and cerebrospinal fluid (CSF) components using the DARTEL method. They were then spatially normalized to the Montreal Neurological Institute (MNI152) standard space. The functional images were also normalized to the same MNI152 template based on their coregistration with the individual T1 anatomical images, using a resampled voxel size of 3 × 3 × 3 mm^3^. (5) Spatial Smoothing: Spatial smoothing was applied using a 6 mm full-width at half-maximum (FWHM) Gaussian kernel (with ReHo and DC values smoothed at the final stage). (6) Detrending: Linear trends were removed from the functional imaging time series. (7) Covariate Regression: Linear regression was used to remove covariates, including the Friston-24 head motion parameters, and signals from white matter and cerebrospinal fluid.

Following the above preprocessing steps, the DPABI software was used for further analysis of the fMRI data. The following brain maps were computed: amplitude of low-frequency fluctuation (ALFF), fractional ALFF (fALFF), regional homogeneity (ReHo), and functional connectivity density (FCD).

Among these, ALFF, fALFF, and ReHo were used to quantitatively evaluate the characteristics of local spontaneous neural activity in each brain region. FCD was employed to reflect the strength of significant functional connectivity between the time series of each voxel and all other voxels across the brain, calculated as the number of connections with correlation coefficients exceeding a preset threshold (r > 0.25). All calculations were performed in individual standard space, using the default settings of DPABI unless otherwise specified.

### 2.5 ALFF and fALFF

For each voxel's time series, a fast Fourier transform (FFT) was applied to convert the signal into a frequency-domain power spectrum. The square root of the power spectrum within the preset frequency band (0.01–0.1 Hz) was calculated, and the average value was taken as the ALFF. The fALFF value was defined as the ratio of the sum of the power spectrum in the target frequency band (0.01–0.1 Hz) to the sum of the power spectrum across the full frequency range (0–0.25 Hz). fALFF helps improve the specificity and sensitivity of detecting local spontaneous brain activity. To eliminate individual global differences, all ALFF and fALFF images were standardized by the global mean within the whole-brain mask, yielding normalized ALFF (mALFF) and fALFF (mfALFF) maps.

### 2.6 ReHo

Kendall's coefficient of concordance (KCC) was used to evaluate the consistency of the time series between each voxel and its 26 neighboring voxels within the whole-brain mask. The resulting ReHo values reflect local synchronization. Subsequently, ReHo images were globally mean-normalized to obtain normalized ReHo (mReHo) maps and spatially smoothed using a 6 mm FWHM.

Functional connectivity density (FCD) analysis. Connectivity Density (FCD) (36, 37) analysis was performed using a custom batch script in MATLAB to efficiently compute global FCD (gFCD) and local FCD (lFCD, within a 6 mm spherical neighborhood). Time series were first bandpass-filtered, followed by correlation analysis between each voxel and all other voxels in the brain, with a threshold of r > 0.6. The FCD results were normalized by mean and standard deviation for further group-level statistical analysis.

### 2.7 ASL data preprocessing

The preprocessing of ASL data was conducted on the MATLAB platform using the ASLtbx (https://www.cfn.upenn.edu/zewang/ASLtbx.php) and SPM12 (http://www.fil.ion.ucl.ac.uk/spm/) toolkits. Following the default workflow of ASLtbx, the raw ASL data were initially subjected to a series of automated corrections, including motion correction and parameter adjustments such as labeling delay. This process generated denoised individual cerebral blood flow (CBF) maps. Subsequent post-processing steps for the CBF maps included: spatial normalization to the MNI152 standard space, resampling to a spatial resolution consistent with the fMRI data (3 × 3 × 3 mm^3^), and spatial smoothing with a 6 mm full-width at half-maximum (FWHM) Gaussian kernel to enhance signal-to-noise ratio. Finally, to minimize inter-subject variability in overall signal intensity and improve comparability across groups, the spatially smoothed CBF maps were normalized by their mean values within a standard gray matter mask.

### 2.8 NVC calculation

The coupling index used here is not new. The across-voxel (global) and across-region (atlas-based) correlation between a BOLD-derived map of neural activity and an ASL-derived CBF map was introduced for CBF-connectivity coupling by Liang et al. (38) and has since been applied in cerebral small vessel disease, end-stage renal disease and Parkinson's disease (22, 28, 32, 33). What is new in the present work is its application to H-type hypertension, not the metric itself. The index assumes that, within an individual brain, regions with higher spontaneous neural activity require and receive proportionally greater perfusion; a lower correlation is therefore interpreted as a looser match between demand and supply.

#### 2.8.1 Global-level NVC calculation

Global NVC was computed based on the whole-brain gray matter mask. Correlation analysis was applied between neural signal metrics-including ALFF, fALFF, ReHo, and FCD maps-and CBFmeasures within the gray matter mask. The primary focus was to examine group differences in the average NVC across the whole-brain gray matter, yielding NVC values such as ALFF/CBF, fALFF/CBF, ReHo/CBF, and FCD/CBF. Group differences were then assessed using two-sample *t*-tests, controlling for covariates including sex, age, education level, and mean framewise displacement (FD) (Table 1).

#### 2.8.2 Regional NVC calculation

To address the potential limitation of global NVC analysis in overlooking local information, regional NVC was also calculated using the Automated Anatomical Labeling (AAL) atlas (https://www.gin.cnrs.fr/en/tools/aal/). Specifically, similar to the global NVC calculation, regional NVC values were computed within each region of the AAL atlas, resulting in ALFF/CBF, fALFF/CBF, ReHo/CBF, and FCD/CBF metrics for each region. Given the multiple comparisons involved in regional analysis, false discovery rate (FDR) correction was applied to control for multiple comparisons, with a corrected significance threshold of *p* < 0.05.

## 3 Statistical analysis

### 3.1 Clinical indicators

Statistical evaluation of demographic characteristics, clinical features, and cognitive scale scores between the HHT group and the control group was performed using SPSS software (version 20.0). For continuous variables, independent samples *t*-tests were applied to normally distributed data, while the Mann-Whitney U test was used for non-normally distributed data. Categorical variables (e.g., gender) were analyzed using the χ^2^ test, with the statistical significance level set at *p* < 0.05.

### 3.2 NVC indicators

Independent samples *t*-tests were used to compare differences in NVC between the HHT group and the control group. For both global NVC (the five whole-brain indices) and regional NVC (all AAL regions), multiple comparisons were controlled with the false discovery rate (FDR) procedure at a corrected threshold of q < 0.05; corrected values are reported in Tables 2–7. No Bonferroni or Gaussian random field correction was applied to the atlas-based NVC analyses. All analyses included age, gender, and years of education as covariates. Mean framewise displacement was included as an additional covariate.

**Table 2:** Group differences in global neurovascular coupling (NVC).

Item	HHT	HC	*p* Raw	*p* FDR	T
ALFF/CBF	−0.024 ± 0.048	−0.028 ± 0.042	0.682	0.808	0.411
fALFF/CBF	0.040 ± 0.054	0.045 ± 0.037	0.602	0.808	−0.523
gFCD/CBF	−0.070 ± 0.081	−0.066 ± 0.091	0.808	0.807	−0.244
lFCD/CBF	0.269 ± 0.059	0.300 ± 0.045	0.005^**^	0.026^*^	−2.862^*^
ReHo/CBF	0.064 ± 0.057	0.081 ± 0.046	0.118	0.296	−1.575

^*^p < 0.05,

^**^p < 0.01,

^***^p < 0.001.

### 3.3 Voxel-level statistics and multiple comparisons correction

Group-level statistical analysis of local CBF, ALFF, fALFF, ReHo, and FCD was conducted at the voxel level. Multiple comparison correction was applied using GRF theory while controlling for gender, age, and mean framewise displacement (FD) head motion parameters. Correction parameters were voxel-level *p* < 0.001 and cluster-level *p* < 0.05, two-tailed. GRF theory is the mechanism by which cluster-level family-wise error (FWE) control is achieved in DPABI; GRF and FWE therefore denote a single correction procedure applied to the voxel-wise maps, not two separate ones.

### 3.4 Correlation analysis

Partial correlation analysis was used to evaluate the relationships between whole-brain NVC, regional NVC, and cognitive scale scores in HHT patients, while controlling for age, gender, and education level. The statistical significance threshold was set at *p* < 0.05. Correlation analyses were limited to brain regions showing significant group differences.

### 3.5 Network attribution

No network-level statistical analysis was performed. For description only, each significant cluster was assigned to the large-scale network with which the majority of its voxels overlapped in the Yeo 7-network cortical parcellation (39). Network names used in the Results and Discussion are therefore descriptive labels for individual clusters and are not the basis of any statistical inference.

## 4 Results

### 4.1 Demographic and clinical data

As shown in Table 1, patients with HHT had significantly higher Hcy load than the controls. The patients also showed significant lower MoCA and MMSE scores. The two groups were comparable on other factors including age, gender, and education (*p* > 0.05).

### 4.2 Group differences in resting-state fMRI metrics

**Figure 1:**
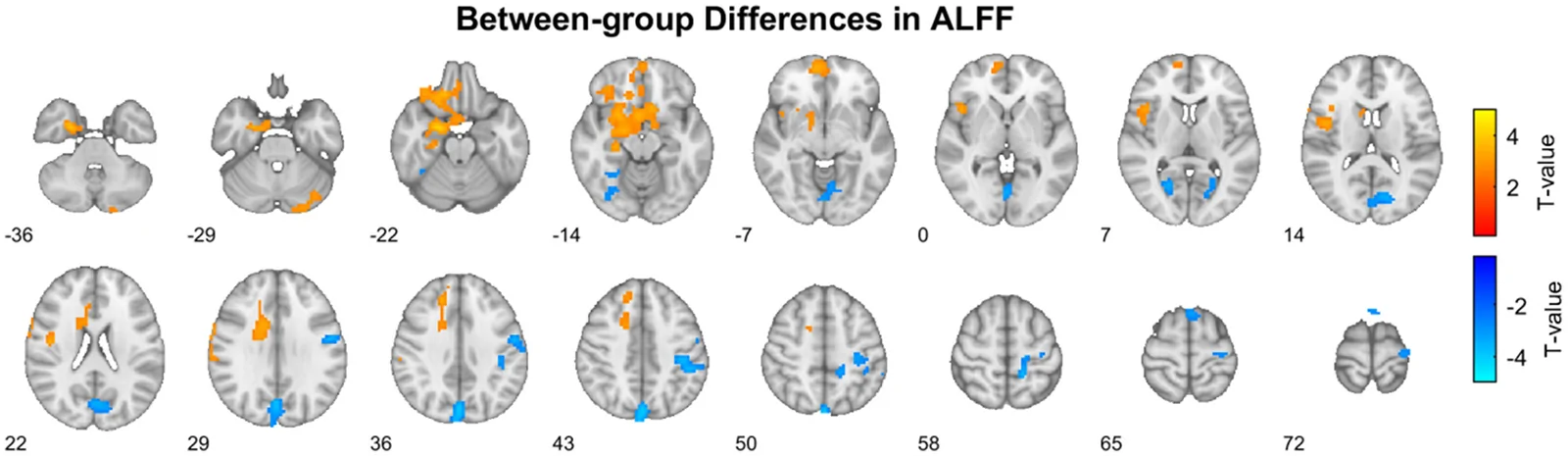
Group differences in ALFF. In the color bar, warm tones indicate higher values in HHT patients compared to controls, while cool tones indicate lower values in patients relative to controls. The colors reflect the *T*-values from the two-sample *t*-test. To ensure visual comparability across different statistical results, the values in the color bar have been standardized. Results are corrected at the voxel level with *p* < 0.001 and at the cluster level with *p* < 0.05, using family-wise error (FWE) correction. Images are displayed in radiological orientation. Note applying to Figures 1–10. All images are presented in radiological orientation, so that the left side of every axial and coronal panel corresponds to the right hemisphere and the right side to the left hemisphere. Anatomical labels are deliberately not overlaid on the statistical maps, which are small multi-slice montages in which superimposed text would obscure the clusters themselves; the full anatomical name, laterality, cluster size, peak T value and MNI coordinates of every significant cluster are instead given in the corresponding table (Table 8 for CBF and Tables 3–7 for the NVC indices), which each figure legend cites. The faint straight lines crossing the sectional panels are the slice-position crosshairs written automatically by the display software and carry no statistical meaning. Where a map is displayed at a more permissive threshold than its table, only the clusters listed in that table survived correction; clusters visible on the map alone are sub-threshold and are not interpreted in the text.

This study first reports the neural activity components involved in neurovascular coupling. Regarding the Amplitude of ALFF, significant group differences were observed in HHT patients, with concurrent increases and decreases in activity within the default mode network. Additionally, increased ALFF was identified in the basal ganglia (Figure 1). The results were corrected at the voxel level with *p* < 0.001 and at the cluster level with *p* < 0.05, using family-wise error (FWE) correction.

**Figure 2:**
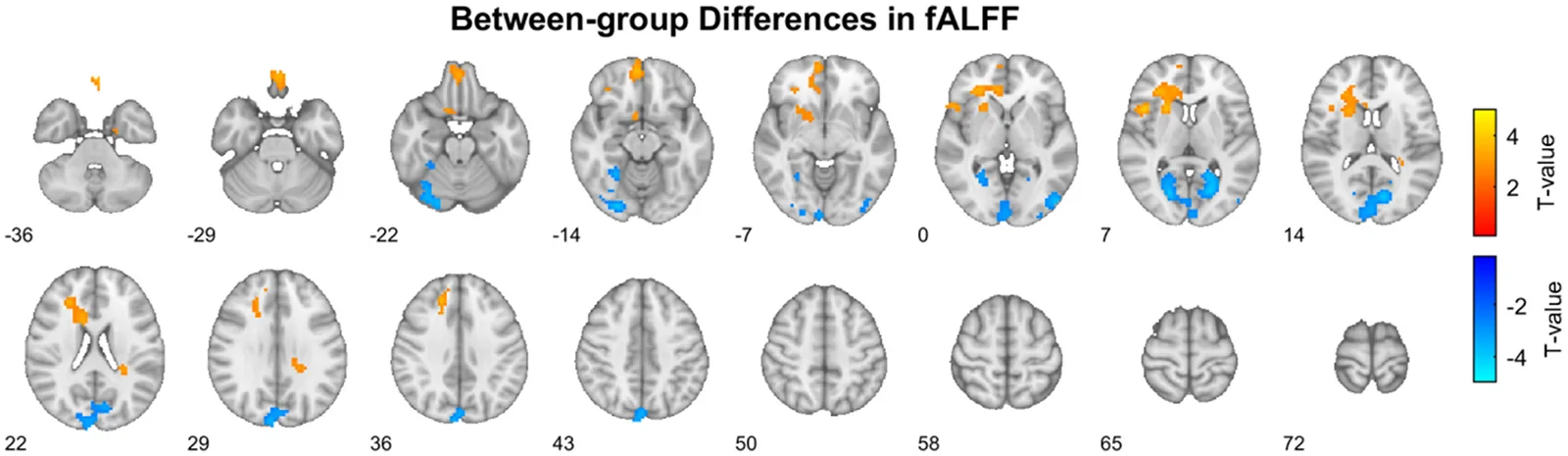
Group differences in fALFF. In the color bar, warm tones indicate that HHT patients have higher values than the control group, while cool tones indicate lower values in patients compared to controls. The colors reflect the *T*-values from the two-sample *t*-test. To ensure visual comparability across different statistical results, the values in the color bar have been standardized. The findings are corrected at the voxel level with *p* < 0.001 and at the cluster level with *p* < 0.05, using family-wise error (FWE) correction. The images are presented in radiological orientation.

This study further analyzed the group differences in fALFF. The advantage of this metric lies in its ability to partially suppress cerebrospinal fluid signals and achieve whole-brain signal normalization, though it correspondingly attenuates useful signals in other frequency bands. In the analysis of this indicator, fALFF was reduced in the posterior part of the default mode network and increased in salience and cognitive control networks (Figure 2).

#### 4.2.1 Global FCD

**Figure 3:**
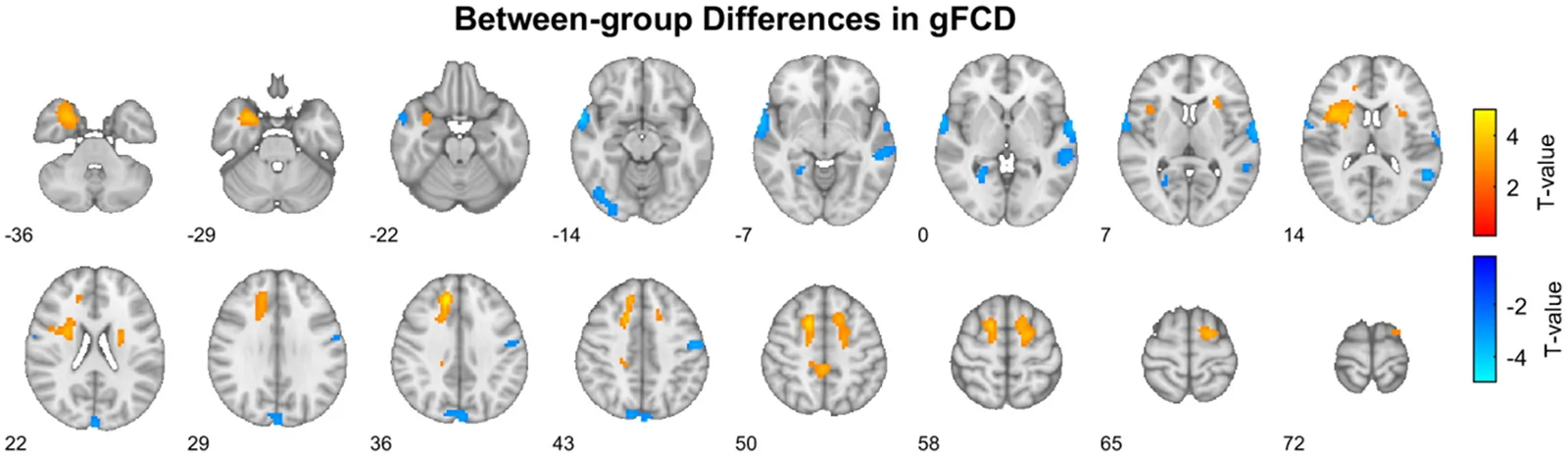
Group differences in global functional connectivity density. In the color bar, warm tones indicate that HHT patients have higher values than the control group, while cool tones indicate lower values in patients compared to controls. The colors reflect the *T*-values from the two-sample *t*-test. To ensure visual comparability across different statistical results, the values in the color bar have been standardized. The findings are corrected at the voxel level with *p* < 0.001 and at the cluster level with *p* < 0.05, using family-wise error (FWE) correction. The images are presented in radiological orientation.

Further analysis based on the AAL3 brain region distribution revealed that gFCD results indicated significantly higher FCD in HHT patients compared to controls in the following regions: the right superior frontal gyrus (area 2), left supplementary motor area, right temporal pole of the middle temporal gyrus, and right middle precentral cortex. Conversely, significantly lower FCD in HHT patients was observed in regions including the right temporal pole of the superior temporal gyrus, left central gyrus, left middle temporal gyrus, right inferior occipital gyrus, left posterior parietal gyrus, and bilateral precuneus (Figure 3).

#### 4.2.2 Local FCD

**Figure 4:**
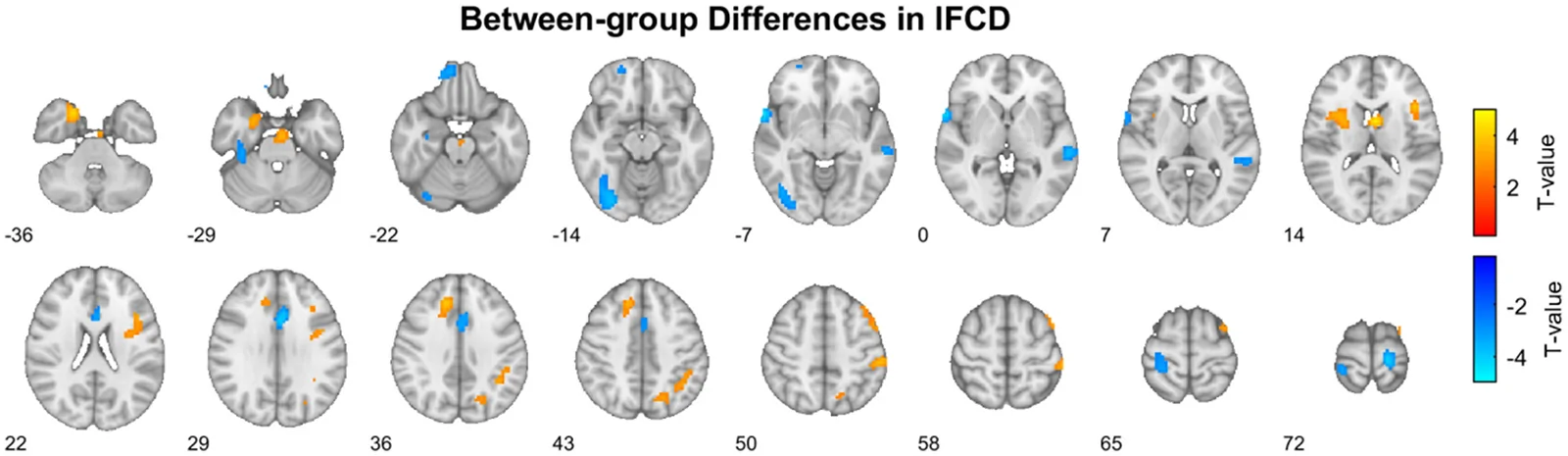
Group differences in local functional connectivity density (lFCD). In the color bar, warm tones indicate that HHT patients have higher values than the control group, while cool tones indicate lower values in patients compared to controls. The colors reflect the T-values from the two-sample *t*-test. To ensure visual comparability across different statistical results, the values in the color bar have been standardized. The findings are corrected at the voxel level with *p* < 0.001 and at the cluster level with *p* < 0.05, using family-wise error (FWE) correction. The images are presented in radiological orientation.

Based on the AAL3 atlas, lFCD analysis revealed that brain regions with significantly higher FCD in HHT patients compared to controls included the left caudate nucleus, right superior frontal gyrus (area 2), left triangular part of the inferior frontal gyrus, left precentral gyrus, right insula, and left inferior and superior parietal lobules. Regions with significantly lower FCD in HHT patients included the right temporal pole of the superior temporal gyrus, left anterior cingulate gyrus, left paracentral lobule, right inferior occipital gyrus, left middle temporal gyrus, right posterior parietal gyrus, right medial orbitofrontal cortex, and right fusiform gyrus (Figure 4).

### 4.3 Group differences in regional CBF

**Figure 5:**
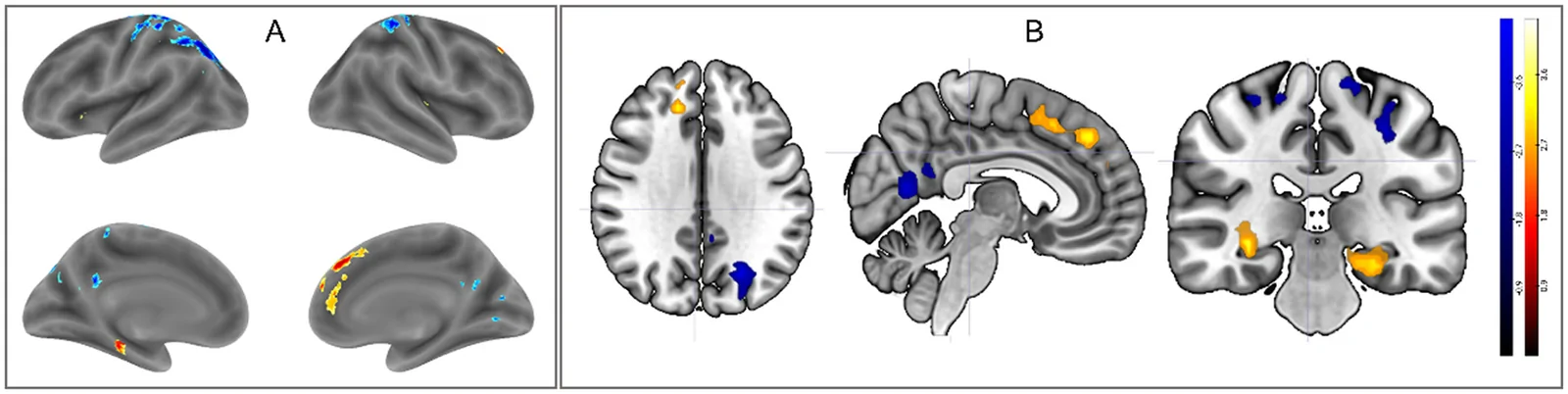
Group differences in regional CBF. **(A)** Cortical surface view. **(B)** Cross-sectional views displaying axial, sagittal, and coronal planes of CBF group differences, presented in radiological orientation. Red-yellow color coding indicates regions where HHT patients show higher CBF compared to controls, while blue color coding indicates regions with lower CBF. Results are corrected at the voxel level with *p* < 0.001 and cluster level with *p* < 0.05 using family-wise error (FWE) correction.

Within the whole-brain gray matter mask, voxel-wise two-sample *t*-tests were performed to assess group differences in regional CBF, while controlling for covariates. Regions with reduced CBF were primarily located in the central sulcus, postcentral gyrus, superior parietal lobule, cuneus, and posterior cingulate cortex. Areas with increased CBF were mainly observed in the supplementary motor area, anterior cingulate cortex, and bilateral hippocampus/parahippocampal gyrus. These findings suggest a hypertension-related redistribution of CBF. Results were corrected at the voxel level with *p* < 0.001 and at the cluster level with *p* < 0.05 using family-wise error (FWE) correction. Images are displayed in radiological orientation, as shown in Figure 5 and Table 8.

### 4.4 Alterations in whole-brain NVC in HHT patients

Following the presentation of group differences in neural activity and CBF, this section focuses on the key metric of whole-brain NVC. As shown in Table 2, whole-brain NVC comprises five indicators. Apart from ALFF/CBF, all other metrics generally demonstrated lower global NVC in the HHT group. Notably, NVC based on local functional connectivity density (lFCD/CBF) exhibited a significant reduction.

### 4.5 Group differences in regional NVC

#### 4.5.1 ALFF/CBF coupling

**Figure 6:**
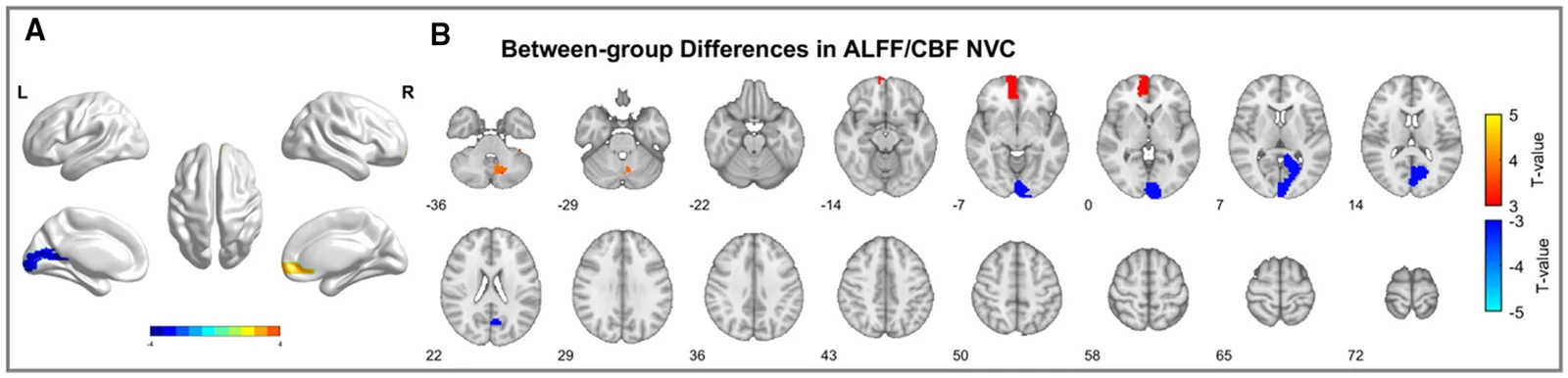
Group differences in regional ALFF/CBF NVC. **(A)** Cortical surface view. **(B)** Cross-sectional view displaying axial plane differences in NVC, presented in radiological orientation. Red-yellow color coding indicates regions where HHT patients show higher NVC compared to controls, while blue color coding indicates regions with lower NVC. Results are corrected at *p* < 0.05, using false discovery rate (FDR) correction.

After FDR correction, altered ALFF/CBF coupling in HHT patients was confined to the left middle occipital gyrus (Figure 6 and Table 3). No other region, including the medial prefrontal and anterior cingulate cortices and the cerebellar cluster visible on the uncorrected map, survived correction for this index; for visualization, Figure 6 is displayed at a more permissive threshold than the table, so that clusters appearing on the map but absent from Table 3 are sub-threshold and are not interpreted.

**Table 3:** Group differences in regional ALFF/CBF neurovascular coupling (NVC).

Brain region	HHT	HC	*T*	*P* Raw	*P* FDR
Left middle occipital gyrus	−0.125 ± 0.076	−0.192 ± 0.103	3.832	0.0002^***^	0.0150^*^

Positive T denotes HHT > HC. Values re-exported from the source statistical output; only regions surviving FDR correction (q < 0.05) are listed. In each table, *p* Raw is the uncorrected *p* value and *p* FDR the Benjamini–Hochberg corrected value (q value); a corrected value can never be smaller than the uncorrected one. ^*^p < 0.05, ^**^p < 0.01, ^***^p < 0.001.

#### 4.5.2 fALFF/CBF coupling

**Figure 7:**
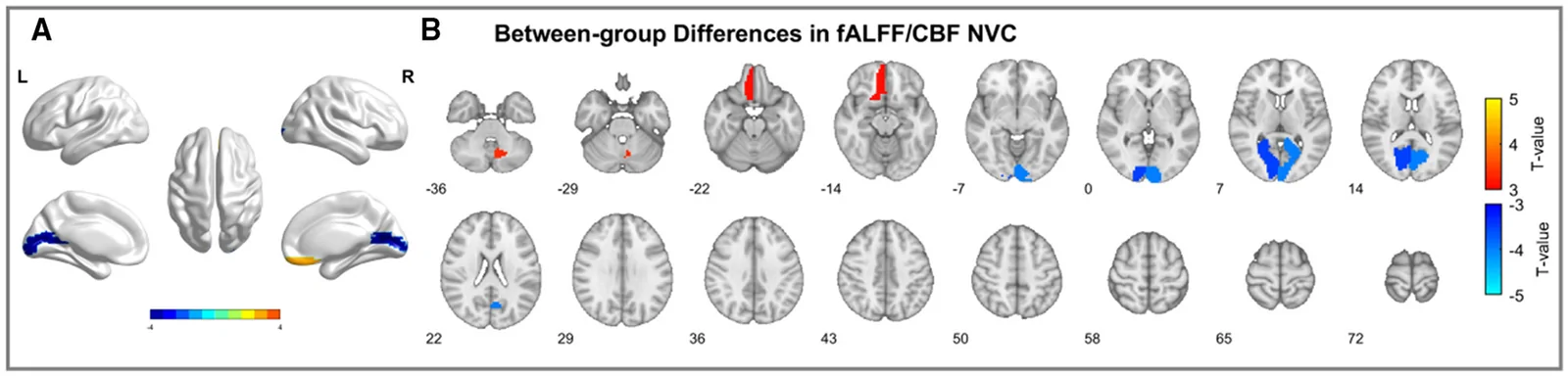
Group differences in regional fALFF/CBF NVC. **(A)** Cortical surface view. **(B)** Cross-sectional view displaying axial plane differences in NVC, presented in radiological orientation. Red-yellow color coding indicates regions where HHT patients show higher NVC compared to controls, while blue color coding indicates regions with lower NVC. Results are corrected at *p* < 0.05 using false discovery rate (FDR) correction.

HHT patients exhibited decreased fALFF/CBF coupling in the right medial superior frontal gyrus, the right amygdala, the left calcarine fissure and right cerebellar lobule X (area 10). All four regions survived correction and all showed reduced coupling in patients; no region showed increased coupling for this index. These results were corrected at *p* < 0.05 using false discovery rate (FDR) correction, as shown in Figure 7 and Table 4.

**Table 4:** Group differences in regional fALFF/CBF neurovascular coupling (NVC).

Brain region	HHT	HC	*T*	*p* Raw	*p* FDR
Right medial superior frontal gyrus	−0.267 ± 0.295	0.440 ± 0.264	−3.131	0.0023	0.0287
Right amygdala	0.416 ± 0.295	0.612 ± 0.173	−3.993	0.0001	0.0061
Left calcarine fissure	0.447 ± 0.319	0.633 ± 0.190	−3.498	0.0007	0.0176
Right cerebellum area 10	−0.104 ± 0.092	0.175 ± 0.124	−3.342	0.0011	0.0196

Positive T denotes HHT > HC. Values re-exported from the source statistical output; only regions surviving FDR correction (q < 0.05) are listed. In each table, *p* Raw is the uncorrected *p* value and *p* FDR the Benjamini–Hochberg corrected value (q value); a corrected value can never be smaller than the uncorrected one.

#### 4.5.3 gFCD/CBF coupling

**Figure 8:**
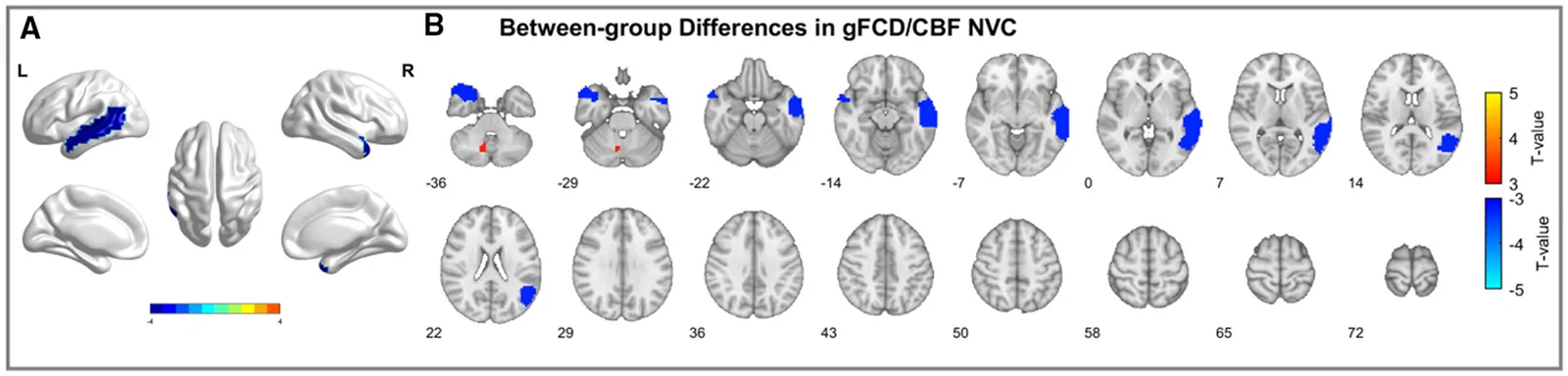
Group differences in regional gFCD/CBF NVC. **(A)** Cortical surface view. **(B)** Cross-sectional view displaying axial plane differences in NVC, presented in radiological orientation. Red-yellow color coding indicates regions where HHT patients show higher NVC compared to controls, while blue color coding indicates regions with lower NVC. Results are corrected at *p* < 0.05, using false discovery rate (FDR) correction.

gFCD reflects long-range connectivity and global integration capacity. gFCD/CBF coupling was reduced in HHT patients in the right inferior temporal gyrus and the left cerebellar vermis (cerebellar declive), and increased in the left cerebellar lobule X (AAL “Cerebelum_10”, rendered as cerebellar tonsil in some atlas look-up tables). The results were corrected at *p* < 0.05, using false discovery rate (FDR) correction, as shown in Figure 8 and Table 5.

**Table 5:** Group differences in regional gFCD/CBF neurovascular coupling (NVC).

Brain region	HHT	HC	*T*	*p* Raw	*P* FDR
Right inferior temporal gyrus	−0.017 ± 0.363	0.204 ± 0.306	−3.309	0.0013	0.0412
Left cerebellar vermis	−0.177 ± 0.214	−0.010 ± 0.309	−3.272	0.0015	0.0155
Left cerebellar lobule X	−0.047 ± 0.128	−0.141 ± 0.167	3.287	0.0014	0.0222

Positive T denotes HHT > HC. Values re-exported from the source statistical output; only regions surviving FDR correction (q < 0.05) are listed. In each table, *p* Raw is the uncorrected *p* value and *p* FDR the Benjamini–Hochberg corrected value (q value); a corrected value can never be smaller than the uncorrected one.

#### 4.5.4 lFCD/CBF coupling

**Figure 9:**
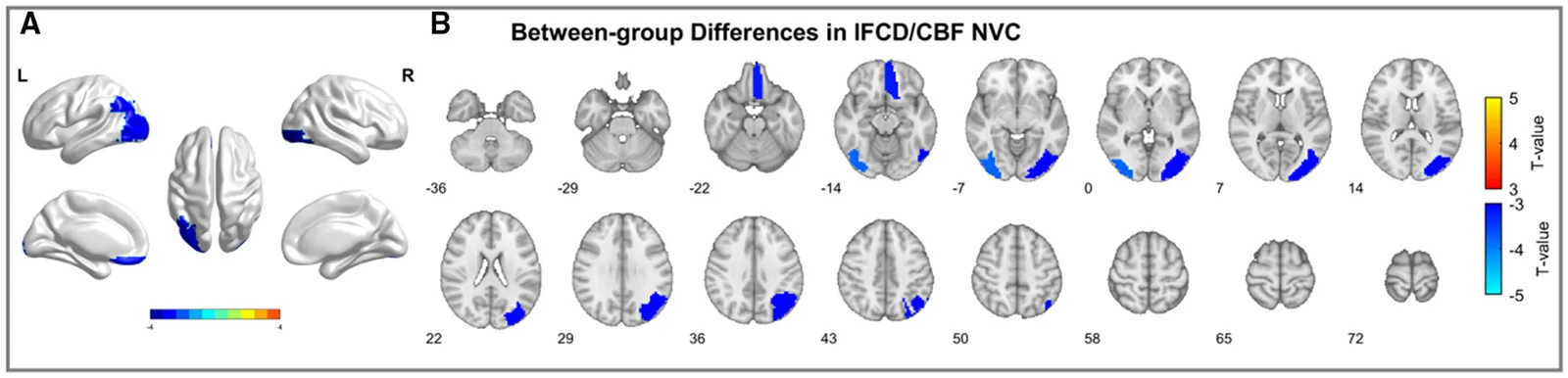
Group differences in regional lFCD/CBF NVC. **(A)** Cortical surface view. **(B)** Cross-sectional view displaying axial plane differences in NVC, presented in radiological orientation. Red-yellow color coding indicates regions where HHT patients show higher NVC compared to controls, while blue color coding indicates regions with lower NVC. Results are corrected at *p* < 0.05, using false discovery rate (FDR) correction.

lFCD reflects short-range connectivity and local segregation ability. After correction, the group difference in lFCD/CBF coupling was confined to the left middle occipital gyrus, where coupling was reduced in HHT patients. Reductions in the right cuneus, the right superior occipital gyrus and the orbital part of the left middle frontal gyrus were present at the uncorrected level but did not survive correction, and are therefore not interpreted here. The result was corrected at *p* < 0.05, using false discovery rate (FDR) correction, as illustrated in Figure 9 and detailed in Table 6.

**Table 6:** Group differences in regional lFCD/CBF neurovascular coupling (NVC).

Brain region	HHT	HC	*T*	*p* Raw	*p* FDR
Left middle occipital gyrus	0.267 ± 0.306	0.475 ± 0.242	−3.7845	0.0134	0.0290

Positive T denotes HHT > HC. Values re-exported from the source statistical output; only regions surviving FDR correction (q < 0.05) are listed. In each table, *p* Raw is the uncorrected *p* value and *p* FDR the Benjamini–Hochberg corrected value (q value); a corrected value can never be smaller than the uncorrected one.

#### 4.5.5 ReHo/CBF coupling

**Figure 10:**
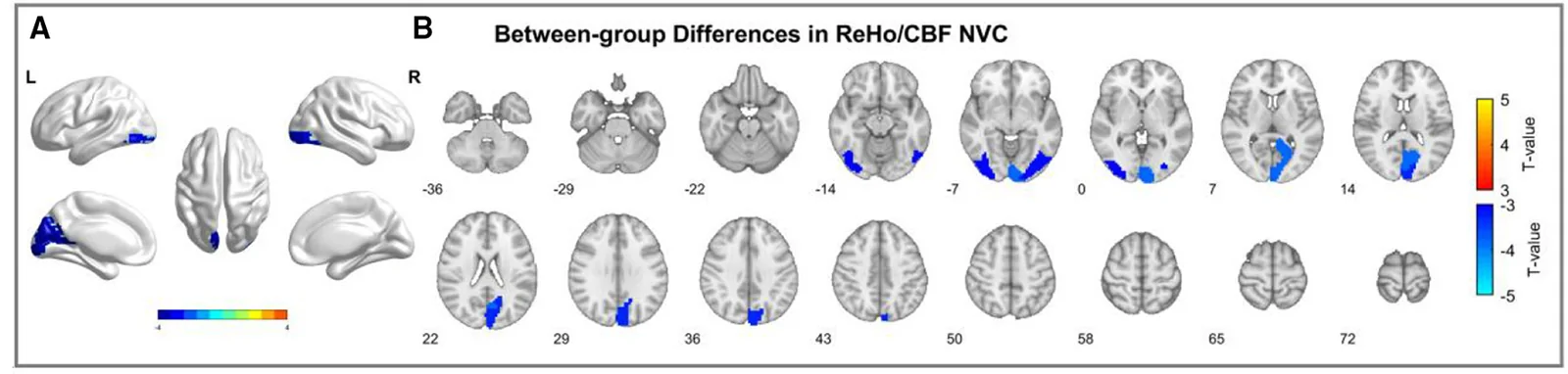
Group differences in regional ReHo/CBF NVC. **(A)** Cortical surface view. **(B)** Cross-sectional view displaying axial plane differences in NVC, presented in radiological orientation. Red-yellow color coding indicates regions where HHT patients show higher NVC compared to controls, while blue color coding indicates regions with lower NVC. Results are corrected at *p* < 0.05, using false discovery rate (FDR) correction at the voxel level.

ReHo reflects short-range connectivity and local segregation ability. After correction, the group difference in ReHo/CBF coupling was confined to the right amygdala, where coupling was reduced in HHT patients. Reductions in the right calcarine fissure and the left middle occipital gyrus did not survive correction and are not interpreted. The result was corrected at *p* < 0.05, using false discovery rate (FDR) correction, as shown in Figure 10 and detailed in Table 7.

**Table 7:** Group differences in regional ReHo/CBF neurovascular coupling (NVC).

Brain region	HHT	HC	*T*	*p* Raw	*p* FDR
Right amygdala	0.629 ± 0.182	0.748 ± 0.120	−3.807	0.0144	0.0268

Positive T denotes HHT > HC. Values re-exported from the source statistical output; only regions surviving FDR correction (q < 0.05) are listed. In each table, *p* Raw is the uncorrected *p* value and *p* FDR the Benjamini–Hochberg corrected value (q value); a corrected value can never be smaller than the uncorrected one.

### 4.6 Correlation analysis

**Figure 11:**
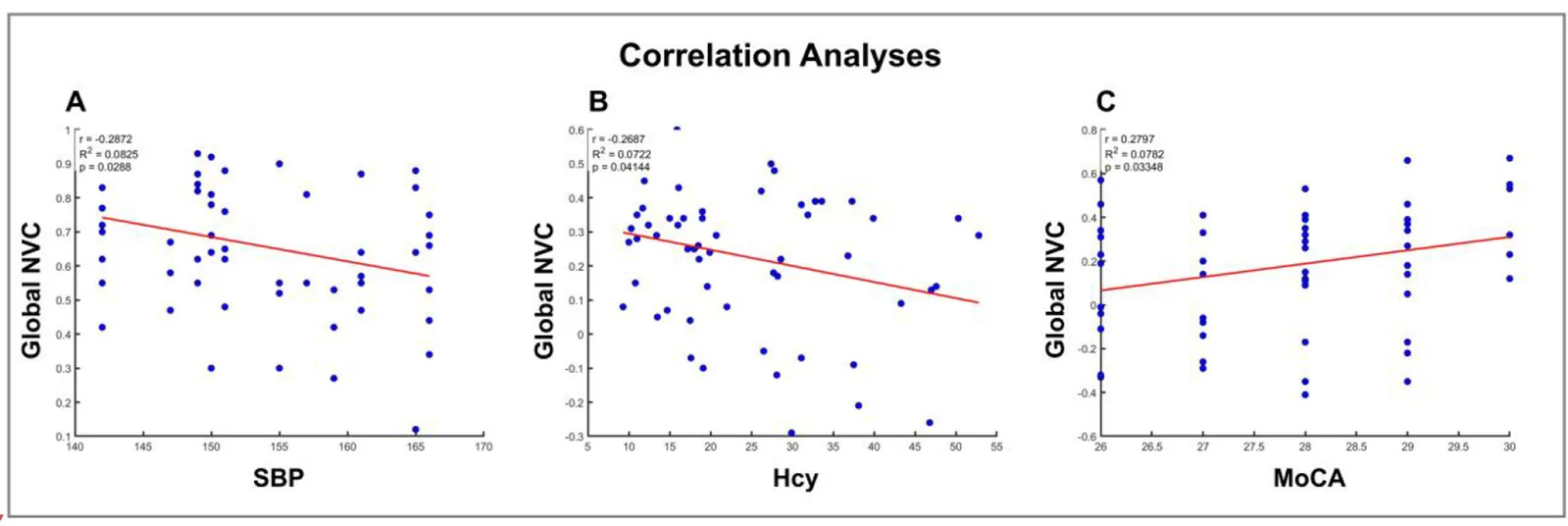
Correlation analysis. **(A)** Scatter plot showing the correlation between whole-brain NVC and SBP. **(B)** Correlation between whole-brain NVC and plasma Hcy levels. **(C)** Correlation between whole-brain NVC and MoCA scores.

To understand whether NVC is associated with other risk factors and cognitive performance, we performed partial correlation analysis, controlling for age, sex and years of education, between the global ReHo/CBF NVC (which showed group differences) and patients' systolic blood pressure (SBP, mmHg), diastolic blood pressure (DBP, mmHg), plasma homocysteine (Hcy) levels, and MoCA and MMSE cognitive test scores. Global NVC was negatively correlated with SBP (r = −0.2872, *p* = 0.0268) and Hcy (r = −0.2687, *p* = 0.0414), and positively correlated with MoCA (r = 0.2797, *p* = 0.03346). By conventional criteria these associations are of moderate strength (Figure 11). The corresponding partial correlations of global NVC with DBP and with MMSE were also examined and did not reach statistical significance (both *p* > 0.05); the narrow range of MMSE scores in this cohort (29.02 ± 1.17 in patients, 29.74 ± 0.53 in controls) leaves little variance for a correlation to detect.

## 5 Discussion

Neurovascular coupling is a fundamental principle underlying human brain function. In the healthy brain, the relationship between neural activity and cerebral blood flow-referred to as NVC—is closely linked and exhibits distinct patterns across different brain regions. This process, which constitutes the core of cerebral autoregulation, involves metabolic demands from neural activity leading to vasodilation and increased blood flow, creating a “mismatch between oxygen consumption and blood supply” and thereby maintaining a dynamic balance of energy expenditure (40–45).

In recent years, the association between spontaneous brain activity measured by resting-state fMRI (as a proxy for regional neural activity) and cerebral blood flow images obtained through ASL (as a proxy for local cerebral perfusion) has been utilized as a derived metric to approximate the measurement of neurovascular coupling (NVC). Studies have consistently demonstrated that alterations in neurovascular coupling-particularly a decline, are associated with cognitive impairment and disease progression (28–33).

The components of the NVU are tightly interconnected, forming an integrated anatomical and functional system that regulates CBF, which is a prerequisite for normal brain and cognitive function. This study investigated alterations in NVC in patients with hypertension and HHT, revealing the relationship between resting-state spontaneous brain activity, functional connectivity, and cerebral blood perfusion. Three main findings emerged, characterizing how neurovascular coupling at the macroscopic brain tissue level differs between patients with HHT and normotensive controls. As the design is cross-sectional, these differences are described as associations throughout.

Within the whole-brain gray matter mask, voxel-wise two-sample *t*-tests were conducted while controlling for covariates to identify group differences in regional CBF. Regions with reduced CBF were primarily located in the central sulcus, postcentral gyrus, superior parietal lobule, cuneus, and posterior cingulate cortex. In contrast, increased CBF was observed in the supplementary motor area, anterior cingulate cortex, and bilateral hippocampus/parahippocampal gyrus. These findings suggest a hypertension-related redistribution of CBF.

The observed changes in regional CBF, involving both increases and decreases, indicate a redistribution of local cerebral blood perfusion across different brain regions. Areas with reduced CBF, such as the central sulcus, postcentral gyrus, superior parietal lobule, cuneus, and posterior cingulate cortex, are primarily associated with two large-scale brain networks: the somatomotor network (SMN) and the default mode network (DMN). The SMN is involved in motor control and somatosensory perception, playing a critical role in executing bodily movements, perceiving somatic sensations, and integrating motor and sensory information. From this perspective, hypertension-related symptoms such as dizziness, headaches, neck stiffness, and fatigue align with the functional roles of the SMN. The central sulcus, postcentral gyrus, and anterior portion of the superior parietal lobule largely belong to the SMN, while the posterior superior parietal lobule serves as a transitional zone between networks, with its posterior portion associated with the dorsal attention network, which is involved in spatial processing and attention. These findings are consistent with previous neuroimaging studies.

The regions in which coupling was reduced were not randomly distributed, and three features they share offer a coherent account of why they should be affected first. Most are posterior, supplied predominantly by the posterior circulation and lying at or near arterial watershed territory, which is the vascular compartment most exposed to a rightward-shifted autoregulatory curve and to a reduced perfusion reserve. They carry among the highest resting metabolic rates and rates of aerobic glycolysis in the human cortex, so that a constrained supply leaves the smallest margin precisely where demand is greatest. And they are the same regions in which our own voxel-wise analysis found reduced CBF (Table 8, Figure 5). The convergence of an independent perfusion finding with the coupling finding in overlapping territory is the strongest internal evidence that the regional pattern reflects a systematic process rather than noise.

**Table 8:** Group differences in CBF.

Brain region	Cluster size	T-value	MNI coordinates		
			x	y	z
Right amygdala	122	3.832	24	−6	−12
Left hippocampus	123	3.942	−30	−24	−15
Right medial superior frontal gyrus	280	3.793	9	39	42
Right supplementary motor area (SMA)	280	3.336	3	12	54
Left putamen	83	3.182	−27	−3	12
Left superior parietal lobule	275	−4.46	−24	−66	39
Left calcarine sulcus	83	−4.004	3	−66	12
Left paracentral lobule	212	−3.847	−15	−33	66
Left postcentral gyrus	212	−3.789	−36	−39	63

The DMN is a prominent focus in neuroscience. It refers to a set of brain regions most active during rest but deactivated during externally oriented attention tasks. Over the past two decades, research has shed light on DMN functions, including self-referential thinking, memory retrieval, future planning, mind-wandering, self-awareness, introspection, social cognition, theory of mind, empathy, and the integration of internal and external information. This study identified the posterior cingulate cortex (PCC) as a core region of the DMN, consistent with numerous large-scale brain network parcellations that classify the PCC as a central DMN component. Neuroimaging studies suggest that the PCC is highly active during rest and spontaneous thinking, playing a crucial role in introspection and self-referential processing (46). The precuneus, another core DMN component, is spatially and functionally closely linked to the PCC. Previous research indicates that the precuneus works in concert with the PCC to support key DMN functions such as spontaneous recall, scene construction, and episodic simulation. In many brain network atlases, the precuneus and PCC are considered inseparable components of the network.

One component of hypertensive brain damage that the present analysis does not capture, but that bears directly on the interpretation, is the enlarged perivascular space (ePVS). ePVS are a recognized marker of impaired perivascular clearance and of small vessel injury in hypertension (47). Rim et al. (48) recently showed that ePVS burden in the white matter, basal ganglia and midbrain is independently associated with resting muscle sympathetic nerve activity (MSNA) in normotensive and hypertensive adults, indicating that perivascular pathology and central sympathetic outflow are coupled rather than independent features of the hypertensive brain. This is relevant to our data because the basal ganglia, where we observed increased ALFF and increased lFCD, is one of the territories in which ePVS burden tracks sympathetic outflow, which offers a plausible link between the microstructural and the functional descriptions of hypertensive brain injury.

The same group reported an inverse relationship between MSNA and regional gray-matter density in the left superior parietal lobule and right cuneus in hypertensive but not in normotensive participants, together with greater gray-matter density in the precuneus in hypertension (49). The overlap with the present findings is partial, but it runs in the expected direction. The left superior parietal lobule and the cuneus were among the regions in which we found reduced perfusion (Section 3.3, Table 8), and reduced lFCD/CBF coupling in the right cuneus and right superior occipital gyrus was present in our data at the uncorrected level, although it did not survive FDR correction; the coupling difference that did survive for this index, in the left middle occipital gyrus, lies in the same posterior territory. The precuneus showed both reduced CBF and reduced gFCD. For the parietal and cuneal cortex the two literatures point the same way: higher sympathetic drive is associated with lower gray-matter density, and in our data with a loss of match between neural activity and perfusion. The precuneus is the more interesting case, since greater gray-matter density there coexists with reduced perfusion and reduced coupling in our cohort. These need not be contradictory, in that early structural change in this region may be reactive or compensatory rather than degenerative; adjudicating between these possibilities requires studies that acquire MSNA, morphometry and coupling in the same participants.

A route by which homocysteine itself could raise sympathetic drive is also plausible. Homocysteine is an NMDA receptor agonist and reaches central autonomic nuclei, including the rostral ventrolateral medulla and the paraventricular nucleus, where NMDA-mediated excitation increases sympathetic outflow; homocysteine-induced oxidative stress lowers nitric oxide bioavailability, removing tonic sympathoinhibition and impairing baroreflex sensitivity; and the resulting endothelial dysfunction reinforces the pressure load in a self-sustaining loop (50, 51). If hyperhomocysteinemia does raise central sympathetic drive, and sympathetic drive is associated with both ePVS burden and regional gray-matter change, then the reduced coupling observed here may reflect a sympathetically mediated pathway in addition to direct endothelial injury. We advance this as a hypothesis consistent with the present data rather than as a demonstrated mechanism, since MSNA was not recorded in this cohort.

HHcy is an independent risk factor for hypertension-related cognitive impairment (7, 8, 50). Hypertension can lead to cognitive decline through multiple mechanisms, including imbalances in cerebral blood perfusion, disruption of the blood-brain barrier (BBB), neuroinflammation, and β-amyloid (Aβ) deposition. HHcy further exacerbates hypertension-induced cognitive impairment and promotes the development of neurodegenerative diseases such as vascular dementia and Alzheimer's disease (7, 10, 52). Potential mechanisms include: 1. Vascular endothelial oxidative damage: HHcy induces oxidative stress in vascular endothelial cells, reducing vascular elasticity and increasing susceptibility to hypertension, thereby worsening cerebral microcirculation. 2. Synergistic neurovascular injury: Elevated homocysteine levels and hypertension act together to impair cerebral blood flow regulation, increase BBB permeability, and intensify neuroinflammation, accelerating Aβ pathology. 3. Cognitive deterioration: Clinical studies have shown that patients with HHcy and hypertension experience significantly faster cognitive decline than those with hypertension alone, particularly in executive function, memory, and information processing speed (10, 52). These findings suggest that HHcy is not only a synergistic risk factor for hypertension but also a key driver of cognitive impairment progression. Interventions targeting HHcy may thus represent an important strategy for preventing and treating hypertension-related cognitive decline.

Several limitations in this study: First, given the rapid advancements in large-sample, multi-center studies and artificial intelligence, the current sample size is relatively limited. Future research should address this by incorporating larger samples, multi-center data, and longitudinal designs. Second, the functional imaging methods used in this study are indirect measures of large-scale brain activity and do not capture direct electrical activity. While direct electrophysiological recordings are challenging in clinical settings, future studies may track special cases under suitable conditions. Additionally, animal experiments could be conducted to enable cross-modal signal acquisition, integration, and analysis. Third, only right-handed participants were enrolled, in order to reduce variability in functional lateralization; the findings, and in particular the lateralized occipital and parietal effects, cannot be assumed to generalize to left-handed or ambidextrous individuals. Fourth, although cognitive testing was carried out by an assessor who took no part in imaging or analysis, complete blinding to group allocation could not be guaranteed. Fifth, enlarged perivascular spaces were not quantified, as the acquisition was not optimized for that purpose; combined ePVS and coupling assessment is the natural next step. Sixth, body weight was not recorded at enrolment, so body mass index could not be calculated or entered as a covariate. The coupling index used here is a within-subject correlation between two globally scaled maps and is therefore relatively insensitive to global anthropometric differences, but the inability to adjust for adiposity remains a limitation.

## 6 Conclusion

Patients with HHT exhibit alterations in both global and local NVC, particularly in components of the somatomotor and default mode networks. Global NVC was also associated with homocysteine levels and with cognitive performance. Because the design is cross-sectional, these results should be read as associations rather than as evidence that HHT causes the observed changes; longitudinal work is required to establish directionality and to determine whether coupling measures carry prognostic value for cognitive decline and future vascular events.

## Data Availability

The original contributions presented in the study are included in the article/supplementary material, further inquiries can be directed to the corresponding author.
